# Loss-of-Function Alleles of *Heading date 1* (*Hd1*) Are Associated With Adaptation of Temperate *Japonica* Rice Plants to the Tropical Region

**DOI:** 10.3389/fpls.2018.01827

**Published:** 2018-12-10

**Authors:** Sung-Ryul Kim, Gideon Torollo, Mi-Ra Yoon, Jieun Kwak, Choon-Ki Lee, G. D. Prahalada, Il-Ryong Choi, Un-Sang Yeo, O-Young Jeong, Kshirod K. Jena, Jeom-Sig Lee

**Affiliations:** ^1^Strategic Innovation Platform, International Rice Research Institute, Metro Manila, Philippines; ^2^Rice Breeding Platform, International Rice Research Institute, Metro Manila, Philippines; ^3^National Institute of Crop Science, Rural Development Administration, Jeonju, South Korea

**Keywords:** *Hd1*, heading date, flowering time, temperate *japonica*, tropics, genetic diversity, rice

## Abstract

Adaptation of temperate *japonica* rice varieties to tropical regions is impeded by extremely early flowering probably due to photoperiod change from long to short. However, constant breeding efforts led to development of temperate *japonica* varieties adapted to tropical/subtropical regions, but the genetic factor underlying this is still elusive. We analyzed the 45 diverse rice accessions and 12 tropical-adapted temperate *japonica* lines for the allele types of seven major flowering genes *Hd1, OsPPR37, DTH8, Ghd7, Ehd1, RFT1*, and *Hd3a* and flowering time under three different field conditions in temperate and tropical locations. The accessions originated from the tropical/subtropical regions preferred the non-functional alleles of *Hd1* and not other flowering genes. The genetic effect analysis of each gene showed that only the functional *Hd1* caused early flowering in the tropical location. All 12 temperate *japonica* breeding lines adapted to the tropics possessed the loss-of-function alleles of *Hd1* with no change of other flowering genes compared to common Korean temperate *japonica* varieties. A phylogenetic analysis using 2,918 SNP data points revealed that the genome status of the 12 breeding lines were very similar to Korean temperate *japonica* varieties. These results indicate that the functional *Hd1* alleles of temperate *japonica* varieties induced extremely early flowering in the tropics and the non-functional *hd1* alleles brought about the adaptation of temperate *japonica* rice to tropical regions.

## Introduction

Rice is a major staple food in the world especially in Asian countries. It is also the most rapidly growing food source in African countries (Seck et al., 2012). Various rice genotypes are cultivated in wide ranges of geographical locations in the world (40° S – equator – 53° N). In temperate zones including China (mainly in the northeast regions), Japan, Korea, Central Asia, and Europe in the northern hemisphere and Australia, Chile, and Uruguay in the southern hemisphere, *japonica* rice types are mostly grown once a year because of the occurrence of frost winter (Jena and Hardy, 2012). In contrast, in tropical zones surrounding the equator (23.2° S – equator – 23.2° N) and in some subtropical zones, *indica* rice types are widely grown in a double-season rice rotation cropping system because of the mild temperature throughout the year.

Rice is a short-day (SD) plant in which flowering is promoted by a reduced day-length. Flowering time (called as heading date in rice) is an important trait of rice for local domestication and is also one of the priority traits in rice breeding programs because it influences rice grain yield (Wei et al., 2010; Endo-Higashi and Izawa, 2011; Murphy et al., 2011; Yan et al., 2011; Gao et al., 2014; Liu et al., 2015) and other traits such as plant architecture and duration of vegetative phase (Xue et al., 2008; Hori et al., 2015) as well as cropping systems (Toriyama, 1970). The adjustment or fine-tuning of the flowering time of rice varieties can bring about some advantages such as enhanced multiple rice cropping per year, precise rotation cropping systems with other profitable crops, high prices of rice for specific consumers, and the possible improvement of rice field management by avoiding a regular encounter with biotic/abiotic stresses.

To date, more than 40 different genes (>40 gene loci) regulating flowering time in rice have been identified by several approaches including map-based cloning, reverse genetics approach using T-DNA or transposon tagging lines, and control of target gene expression via RNAi and overexpression. Most of the genes were identified and studied in high-latitude field conditions [natural long-day (LD)] or in day-length controlled chambers (Lee and An, 2015a; Hori et al., 2016). Furthermore, intensive research using physiology, genetics, and molecular biology tools quite revealed the molecular mechanisms of rice flowering. The regulatory networks among the flowering-associated genes are monitoring environment factors (mostly day-length in rice) and eventually activating the expression of the florigen genes *Heading date 3a* (*Hd3a*) and/or *RICE FLOWERING LOCUS T 1* (*RFT1*) for rice plants to flower. Both Hd3a and RFT1 are mobile proteins that eventually cause the transition from the vegetative shoot apical meristem to the reproductive meristem which is called panicle initiation in rice (Tamaki et al., 2007; Komiya et al., 2008). *Hd3a* and *RFT1* are paralogous to each other with 91% amino acid homology and are very closely located to each other with a physical distance of ∼11 kb on the chromosome 6. The expression of the florigen genes is tightly regulated by several upstream genes. *Heading date 1* (*Hd1*) encoding a homolog of Arabidopsis *CONSTANS* promotes flowering by activating the transcription of the florigen genes under SD condition, but it suppresses the florigen genes under LD condition (Yano et al., 2000; Hayama et al., 2003). *Early heading date 1* (*Ehd1*) encoding a B-type response regulator protein promotes the expression of *Hd3a* and *RFT1* under both SD and LD conditions (Doi et al., 2004). *Grain number, plant height, and heading date7* (*Ghd7*) encoding a CCT domain protein represses the expression of *Ehd1* in LD condition and its functional alleles delay flowering in LD condition (Xue et al., 2008). *DTH8* (QTL for days to heading) encoding the NF-Yb subunit of the trimeric NF-Y transcription factor also delays flowering in LD condition by suppressing the expression of *Ehd1* (Wei et al., 2010) while its allelic mutant *ds9* exhibited early flowering in LD and delayed flowering in SD, respectively (Xing et al., 2015). Another upstream gene of *Ehd1, OsMADS51*, is a flowering activator in SD with major effect and in LD with minor effect (Kim et al., 2007). Recent study by Chen et al. (2018) identified that *OsMADS51* is also involved in high temperature-induced flowering of rice. *Oryza sativa Pseudo-Response Regulator 37* (*OsPRR37*)/*Hd2* also known as *Ghd7.1* (Liu et al., 2013) functions as a flowering suppressor in both SD and LD conditions independently of *Hd1* and *Ehd1* pathways (Koo et al., 2013). The alleles of the identified flowering genes, especially natural variations, need to be more actively utilized in rice breeding programs to optimize the flowering time of local rice cultivars.

The *indica* rice cultivars were extensively utilized for the breeding of high-yielding lines in the temperate zone through shuttle breeding between the International Rice Research Institute (IRRI), Philippines (14.2° N) and the Rural Development Administration (RDA) of Korea. As a consequence, Tongil-type cultivars derived from the *indica* x *japonica* cross were successfully developed and brought the Green Revolution to Korea in the 1970s (Kim et al., 2012). The reverse adaptation of rice cultivars (from temperate zones to the tropics) is less known. In the 1930s, *japonica* rice breeding was conducted in Taiwan (22°N∼25°N) located at the boundary between the subtropics and tropics (called Tropics of Cancer) using Japanese temperate varieties which led to the development of the variety Taichung 65 that had long basic vegetative growth periods; since then, this variety has been widely utilized in *japonica* rice breeding programs in Taiwan (Wasano, 1982; Hsing, 2014). Another breeding program, the Germplasm Utilization Value Added (GUVA) project has initiated to breed high quality and high-yielding temperate *japonica* rice varieties in the tropics at IRRI since 1992. For several years at the beginning of the project, most of the Korean temperate *japonica* varieties were tested at IRRI fields to observe their performance. However, no varieties were found to be adequate for practical cultivation in the tropics mainly because of their extremely early flowering due to reduced day-lengths (Kang, 2010). Nonetheless, a handful of breeding lines showing good performance at IRRI were developed through the conventional breeding methods without recognition of the genetic factors associated with extreme early flowering. Among them, four lines were registered as temperate *japonica* rice varieties in the Philippines. However, the genetic factors influencing the adaptation of temperate *japonica* to tropical and subtropical environments are unclear.

In this study, the phenotypes of temperate *japonica* varieties in the tropical environment of the Philippines were examined and the key gene for a geographical adaptation was elucidated through allele typing of the major heading date genes and observation of flowering time in three different natural field conditions. Furthermore, the use of allele types of the major flowering genes for the fine-tuning of heading date in local rice varieties was discussed.

## Materials and Methods

### Plant Materials

A total of 45 diverse rice accessions were chosen for this study, including 20 IRRI diverse core collections (McNally et al., 2009), temperate *japonica* varieties from Japan, Korea, and Taiwan, *indica*-genome based Tongil-type cultivars of Korea, and popular *indica* varities developed by IRRI, Philippines. These 45 accessions covered the diverse geographical origins and four different rice genotypes including *aus, indica*, tropical *japonica*, and temperate *japonica* (Supplementary Table S1). In addition, 12 temperate *japonica* breeding lines/varieties developed by the GUVA project of IRRI-RDA collaborations in the Philippines were included (Supplementary Table S2). Briefly, in every winter season of Korea, many genetic materials including Korean varieties, germplasm and breeding lines were sent to the Philippines and grown at the IRRI field for seeds multiplication and advancing breeding cycles. The temperate *japonica* lines showing good performance in the Philippines were continuously selected and the lines were further improved by conventional breeding processes including crossing with other *japonica* or *indica* accessions (Kang, 2010; Ha et al., 2011). Since the initiation of the GUVA project in 1992, four breeding lines (IRRI 142, IRRI 152, IRRI 157, and IRRI 202) that have high grain quality and improved grain yield (>4.5t/ha) were approved as *japonica* rice varieties by the National Seed Industry Council of the Philippines and released in the Philippines.

### Testing Locations

All the 57 accessions were tested at Los Baños, the Philippines and Suwon, Sotuh Korea. Los Baños lies in the tropical zone with latitude 14.2° N, 121.2° E and an altitude of 23 m where rice is cultivated generally in two cycles per year, dry season (DS) and wet season (WS), because of yearly mild temperature. In contrast, Suwon, with latitude 37.2° N and 126.6° E and an altitude of 37 m, lies in the temperate region where rice is grown in only one cultivation cycle or a short-duration rice variety with other crops per year because of frost winter.

### Plant Growth

For testing in the low-latitude tropical climate, all the 57 accessions were grown at the IRRI fields (for two regular cropping seasons in the DS and WS) following the standard IRRI rice cultivation protocol. Dates of seeding/transplanting were on November 22, 2016/December 15, 2016 for the 2017 DS and May 29, 2017/June 20, 2017 for the 2017 WS. For each accession, the plants were transplanted at one plant per hill with 20 cm × 20 cm spaces and a total of five rows with 25 hills per row. Fertilizers (t/ha) were applied at a rate of 120-17.5-33-5 (N-P-K-Zn) in the 2017 DS and 90-13-25-5 (N-P-K-Zn) in the 2017 WS according to the seasonal and local standard fertilizer methods. For testing in the high-latitude temperate climate, all materials were grown at the RDA field of Suwon station in Korea according to the local normal rice cultivation method. The accessions were seeded in a seedling bed on April 30, 2016 and the seedling plants were transplanted in the field on May 30, 2016. Transplanting space was 30 cm × 15 cm among hills (one plant per hill) and five rows with 26 hills per accession. The rate of fertilizer applied was 90-45-57 (N-P-K).

### Phenotype Measurement

Days to heading (DTH) is defined by the days from seeding to heading in 50% of total plants. The temperate *japonica* varieties under tropical conditions showed very irregular flowering among plants as well as among tillers in a single plant. They also frequently showed extremely early flowering in the main tiller (∼45 days from seeding). In this study, the DTH for the secondary tillers emerged after the main tiller was measured. The culm length and the grain yield were measured from the 10 Korean *japonica* varieties at the IRRI field in the 2017 DS. The average culm length was calculated from those of 10 plants and grain yield in paddy (t/ha) was calculated from those of 50 plants when the seed moisture was 14%. The phenotype data of the 10 Korean *japonica* varieties in Korea were obtained from the rice variety information released by the RDA. Grain yield in paddy (t/ha) in Korea was deduced by multiplying the yield of milled rice by 1.28^1^.

### Allele Determination of the Heading Date Genes

Leaf tissues were collected at the IRRI field and genomic DNAs were extracted using the modified CTAB method (Kim et al., 2011). Several sets of PCR primers for each gene were designed for the amplification of the protein-coding regions of the genes *Hd1, OsPRR37, DTH8*, and *Ghd7* and the promoter region of the *Hd3a*. For identification of the known functional nucleotide polymorphism (FNP) of *Ehd1* and *RFT1* genes, the FNP containing region was amplified by PCR. PCRs were conducted by using the designed primers for each gene from the 57 rice accessions. The PCR products were then directly sequenced through the Sanger method-based sequencing using the AB3730xl DNA analyzer (Applied Biosystems^2^) by Macrogen^3^. Obtained sequences were analyzed using a web-based BLAST tool^4^ and the BioEdit software^5^. The polymorphic nucleotide positions among accessions were confirmed by manual checking of the sequencing chromatogram files. A large insertion/deletion on the heading date genes (*Hd1, Hd3a*, and *OsMADS51*) was determined by PCR amplification. The primer positions mapped on the gene structure and its sequences are presented in Supplementary Figure S1 and Supplementary Table S3, respectively.

### Phylogenetic Analysis

Genome-wide genotyping was conducted using the Illumina Infinium 6K SNP chip (Thomson et al., 2017) at the IRRI Genotyping Service Laboratory^6^ according to the manufacturer’s instruction. The produced SNP genotype data were further processed in Microsoft Excel software to remove the meaningless SNP data points including monomorphic SNPs, rare allele SNPs, high frequency of heterozygous SNPs, or no data calling. Finally, the phylogenetic tree was constructed using PowerMarker software (version 3.25) (Liu and Muse, 2005) with 2,918 SNP data points. The similarity distance matrix of the alleles was measured using the C.S. Chord 1967 method (Cavalli-Sforza and Edwards, 1967) while the gene distribution tree was constructed using the neighbor-joining method.

## Results

### Performance of the Temperate *Japonica* Rice Varieties in the Tropical Location

**FIGURE 1:**
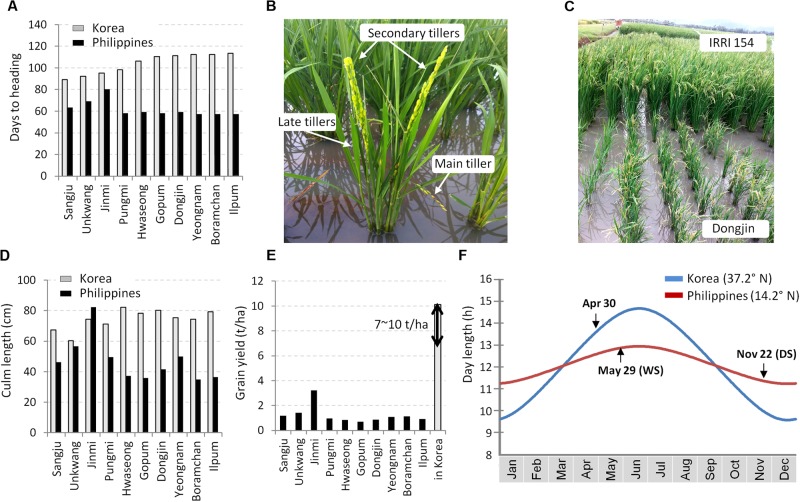
Phenotypes of the temperate *japonica* varieties in the tropical region. Ten Korean temperate *japonica* varieties were tested in the dry season in Los Baños (14.2° N), Philippines and in Suwon (37.2° N), Korea. **(A)** Comparison of days to heading (DTH). **(B)** Irregular flowering among tillers (Dongjin variety). **(C)** Photo of the maturation stage. **(D)** Comparison of culm length. **(E)** Comparison of grain yield. Yield data in Korea were obtained from the rice variety information released by the RDA. **(F)** Seasonal day-length (h) of the RDA Suwon station and the IRRI headquarter in Los Baños. The dates of seeding are depicted in the graph. DS, dry season; WS, wet season.

We grew 10 Korean temperate *japonica* varieties at the IRRI field in the Philippines in DS of 2017. The remarkable phenotypic difference of the varieties between in Korea and in the Philippines was the heading date. Except for Jinmi and Unkwang, eight varieties showed extremely early flowering (<63 DTH) (Figure 1A). These eight varieties exhibited a tendency of irregular flowering time among plants as well as among tillers in a single plant: the embryo-derived main tiller flowered very quickly (∼45 days) and the following tillers flowered in order (Figure 1B). Eventually, these plants became stunted at the reproductive stage (Figures 1C,D) and showed very low grain yield (Figure 1E). As observed, quite early flowering of the elite temperate *japonica* varieties in the tropical zone is a major constraint in breeding temperate *japonica* rice varieties for the tropics. This might have been caused by the reduced day-length according to the geographical transition from a high-latitude location, Korea, to a low-latitude location, the Philippines (Figure 1F). In the Philippines, most of the days in a year belong to the SD condition with a range of day-length from 11.2 to 13 h. In contrast, the basic vegetative phase of rice plants in Korea is under the LD condition (May and June: 13.6 ∼ 14.6 h).

### Allele Typing of the Major Flowering Genes From the 45 Diverse Rice Accessions

**FIGURE 2:**
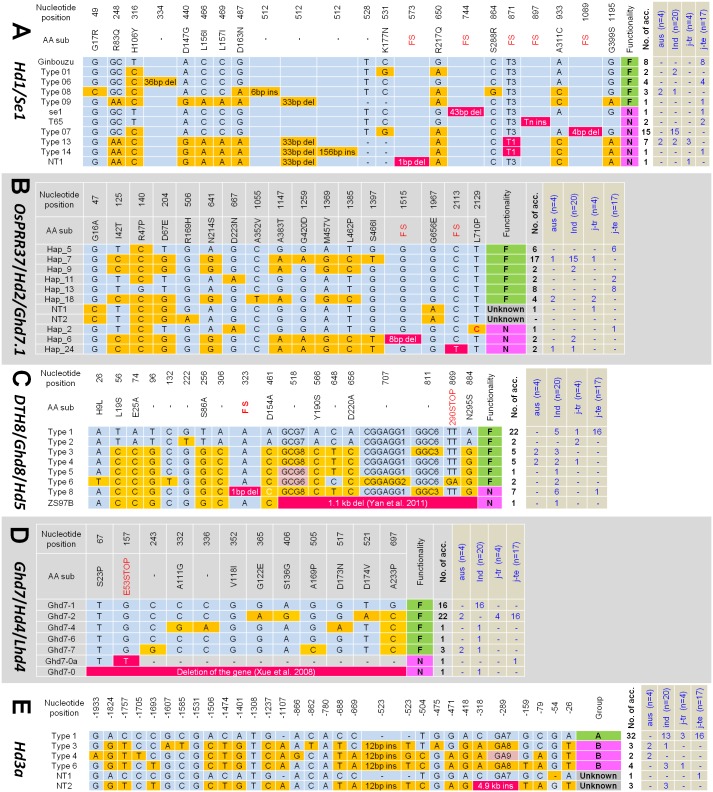
Allele types of the five major flowering genes in the 45 diverse accessions. The allele frequencies in each rice type are presented at the right panel (blue letter). The nucleotide position referred to the protein coding sequence (CDS) or promoter sequence. **(A)** *Hd1* alleles. Reference: Ginbouzu (GenBank Accession No. AB041840.1). **(B)** *OsPRR37* alleles. Reference: Nipponbare (MSU ID: *Os07g49460*). **(C)** *DTH8* alleles. Reference: Nipponbare (MSU ID: *Os08g07740*). **(D)** *Ghd7* alleles. Reference: Minghui 63 (GenBank Accession No. EU286801.1). **(E)** *Hd3a* promoter alleles. Reference: Nipponbare (MSU ID: *Os06g06320*). FS, frame shift; ind, *indica*; j-tr, tropical *japonica*; j-te, temperate *japonica*.

To elucidate the genes causing extremely early flowering in the tropics, the allele types of the major heading date genes were analyzed for the 45 diverse rice accessions including 17 temperate *japonica*, 4 tropical *japonica*, 4 *aus*, and 20 *indica* varieties from different geographical origins (Supplementary Table S1). We sequenced the full coding regions of *Hd1, OsPRR37, DTH8*, and *Ghd7*, and the 2-kb promoter region of *Hd3a* (Supplementary Figure S1). In addition, the PCR products containing the known FNP of the *Ehd1* (G/A SNP causing G221R; Saito et al., 2009) and *RFT1* (G/A SNP causing E105K; Ogiso-Tanaka et al., 2013) were sequenced. Sequences of the genes showed that most allelic variations occurred at the nucleotide positions previously reported. Therefore, the classification of allele types and their functionalities followed those described by Takahashi et al. (2009) for *Hd1* and *Hd3a* promoter, Gao et al. (2014) for *OsPRR37*, Wei et al. (2010) for *DTH8*, and Lu et al. (2012) for *Ghd7*. However, we found new alleles in *Hd1, OsPRR37*, and *Hd3a* and named them as ‘NT (new type).’ In total, 11 allele types of *Hd1*, 10 of *OsPRR37*, 8 of *DTH8*, and 7 of *Gdh7* were found from the 45 accessions (Figures 2A–D). Each allele type was divided into two groups in terms of protein functionality: functional and non-functional alleles. At first, we examined the presence of the prevalent allele types or allele combinations of heading date genes in the tropical location-adapted varieties. Regardless of rice types, most accessions had functional alleles for *OsPRR37* (40 accession), *DTH8* (37 accession), *Ghd7* (43 accession), *Ehd1* (43 accession) and *RFT1* (39 accession), suggesting that these genes might not be the major determinants of flowering time which discriminates between plants grown in temperate zones and in the tropics. In the case of the *Hd3a* promoter, Type 1 allele among the six types was the most frequent in *indica* (13 out of 20), tropical *japonica* (3 out of 4), and temperate *japonica* (16 out of 17) rice types while the other allele types were very rare (Figure 2E). The new allele *Hd3a*-NT2 found in IR8, Saducho, and Zhenshan 97B possessed an insertion of a mobile DNA sequence (∼4.9 kb) near the translation start codon (-292 bp from ATG) (Supplementary Figures S1, S2). However, a certain level of allele prevalence was found for only *Hd1*. The non-functional *hd1* alleles were more frequently found in the tropical/subtropical-adapted accessions (*indica, aus*, and tropical *japonica* types) (23 out of 28). In contrast, the temperate *japonica* varieties mostly possessed the functional alleles of *Hd1* (13 out of 17). Furthermore, we examined the regulatory region of *Hd1* using PCR method because the non-expressed *Hd1* allele caused by insertion of a mobile DNA element at the regulatory region was found (Goretti et al., 2017). However, none of the lines tested harbored the mobile DNA element at *Hd1* promoter region (Supplementary Figure S3).

### Genetic Effects of Individual Flowering Genes in Natural SD and LD Conditions

**FIGURE 3:**
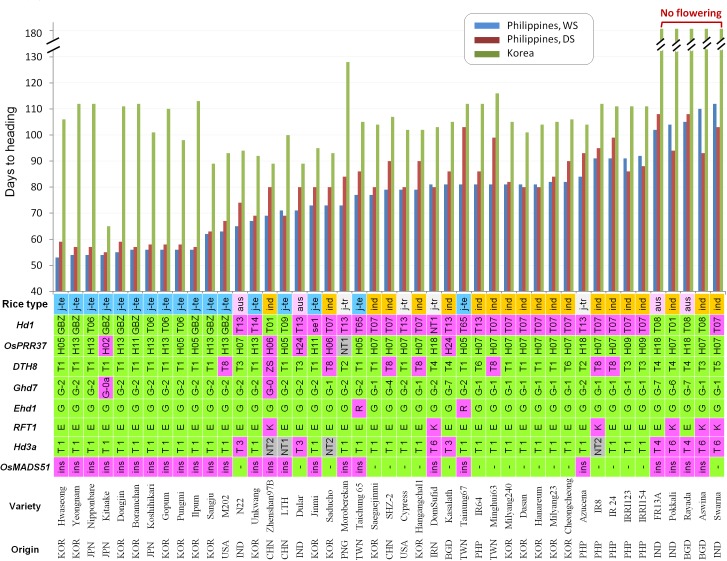
Days to heading (DTH) and the allele types of the major seven heading date genes from 45 diverse accessions. DTH was observed in the three different environments and the samples were sorted by DTH from early to late heading at the IRRI field in the wet season. In the cases of the *Ehd1* and *RFT1*, the FNPs are presented with the corresponding amino acid (G221R of Ehd1 and E105K of RFT1). The tolerance allele to high temperature-induced flowering caused by 9.5-kb insertion at 1^st^ intron of *OsMADS51* is presented by ‘ins.’ The allele types with their functionality to each gene are depicted (green: functional allele and pink: non-functional allele). In the case of the *Hd3a* promoter, Group A and Group B are colored with green and pink, respectively, according to the previous research (Takahashi et al., 2009). New allele types (NT) with unknown functionality are shown with gray color. j-te, temperate *japonica*; j-tr, tropical *japonica*; ind, *indica*.

We examined flowering time of the 45 varieties in the natural SD condition at the IRRI field in the Philippines for two cropping seasons, DS and WS, as well as in the natural LD condition at the RDA field in Korea. Because rice is a SD plant, all 45 varieties were found to flower earlier in natural SD than in natural LD without exceptions (Figure 3). This result supports the fact that rice is a SD plant regardless of types and geographical origins. However, the degree of early flowering was most significant for the Korean temperate *japonica* varieties and three Japanese temperate varieties Nipponbare, Koshikari, and Kitaake at the IRRI field. To examine the genetic effects of each flowering gene, all the accessions were divided into functional and non-functional allele groups for each gene, and the mean values of DTH between these two groups in three different environments were compared (Supplementary Figure S4). The functional alleles of *Hd1* and *RFT1* significantly reduced DTH only in the natural SD conditions (both DS and WS). Except for this, no significant genetic effect of the flowering genes was observed in all three environments.

**FIGURE 4:**
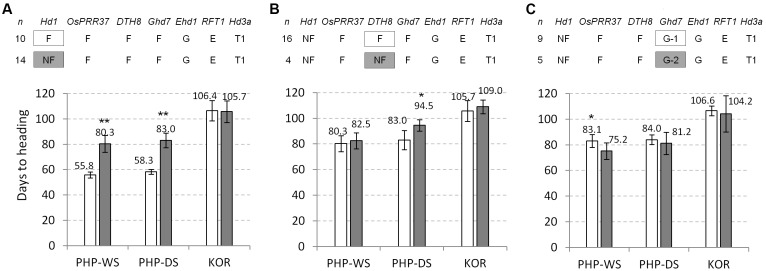
Genetic effects of the major flowering genes on flowering time in three different field conditions. Allele types of each flowering gene and the number of samples are presented on the top panel. **(A)** Genetic effect of *Hd1*. **(B)** Genetic effect of *DTH8*. **(C)** Comparison of the genetic effects between *Ghd7-1* (G-1) and *Ghd7-2* (G-2) alleles. A significant difference between two groups was calculated based on Student’s *t*-test (^∗^*α* = 0.05 and ^∗∗^*α* = 0.01). PHP, Philippines; KOR, Korea. DS, dry season; WS, wet season.

For the more critical evaluations of the flowering genes, the genetic effects were re-analyzed under the same genotype backgrounds of the major flowering genes tested. The group of the functional *Hd1* alleles (*n* = 10) showed quite early flowering in natural SD conditions (24.5 days in WS and 24.7 days in DS) than the group of the non-functional *hd1* alleles (*n* = 14) under the same genotype background: the functional alleles for *OsPRR37, DTH8, Ghd7, Ehd1, RFT1* genes and *Hd3a*-Type 1 allele (Figure 4A). However, between the two groups, there was no difference in flowering time in natural LD. These data indicate that *Hd1* is a strong flowering activator in natural SD but its effect is insignificant in natural LD. Similar analysis showed that the group of non-functional *dth8* alleles (*n* = 4) flowered later than the group of functional *DTH8* alleles (*n* = 14) in all three environments (2.2, 11.5, and 3.3 days in WS, DS, and natural LD, respectively) (Figure 4B). The *Ghd7-2* allele is known as a mild allele compared to the *Ghd7-1* allele (Xue et al., 2008), thus the effect of these two alleles on flowering time within the accessions having the non-functional *hd1* alleles was compared. The plants with *Ghd7-1* allele (*n* = 9) flowered significantly later than those with the *Ghd7-2* allele (*n* = 5) in SD of WS but there was no difference in SD of DS and LD conditions (Figure 4C), suggesting that the two alleles are not much different to each other in terms of the regulation of flowering time. The effects of other gene/allele combinations were not analyzed because of the limited number of samples (less than 3).

### Examination of Temperature-Response Flowering Gene of Rice

**FIGURE 5:**
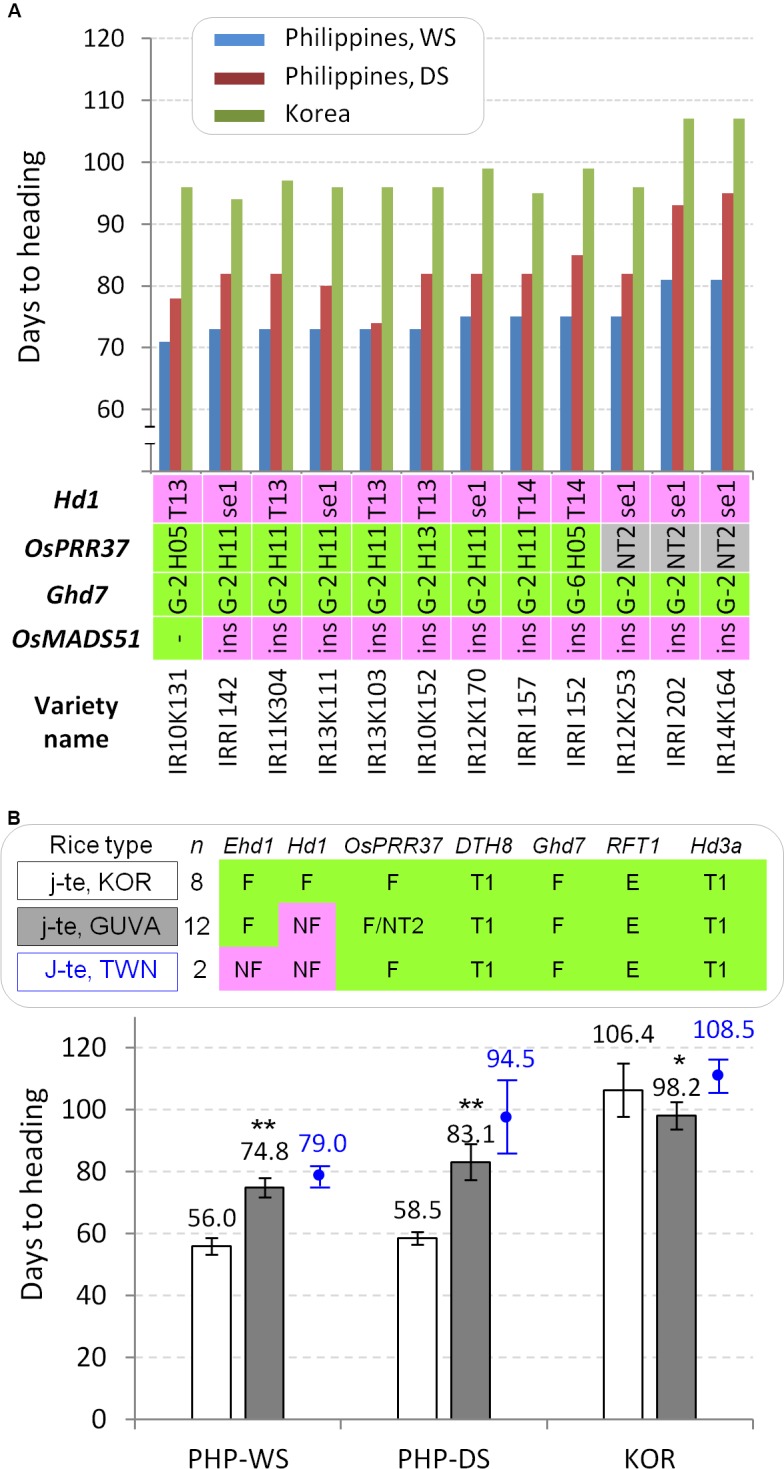
The 12 GUVA temperate *japonica* lines adapted to the tropics. **(A)** DTH and allele types of each flowering gene. Varieties/lines are sorted by DTH at the IRRI in the wet season. All 12 lines have *DTH8*-Type 1, functional *Ehd1*, functional *RFT1*, and *Hd3a*-Type 1 alleles. **(B)** Comparison of DTH between Korean temperate *japonica* varieties (j-te, KOR) and the 12 tropical zone-adapted temperate *japonica* varieties (j-te, GUVA) in three environmental conditions. Student’s *t*-test (^∗^ *α* = 0.05 and ^∗∗^ *α* = 0.01). As references, two Taiwanese temperate *japonica* varieties Taichung 65 and Tainung 67 (j-te, TWN) were presented (labeled by blue color).

Temperature is also considered as a major environmental factor in plant flowering although the clear genetic factors have not been demonstrated yet in rice. Recently, the day-length independent flowering QTL, *qHd1* encoding OsMADS51 showed reduced heading date in response to increased temperature (Chen et al., 2018). A large insertion (∼9.5 kb) at the first intron of *OsMADS51* significantly reduced gene expression and caused tolerance to high temperature-induced flowering. Overall a temperature during cropping season was higher in the tropics than in the temperate region (Supplementary Figure S5). We performed PCRs to identify the presence/absence of the large insertion from the 45 diverse accessions and the 12 *japonica*-type breeding lines adapted to the tropics (Supplementary Figure S6). Interestingly, all 17 temperate *japonica* accessions already possessed the thermo tolerance allele having a large insertion (Figure 3). Similarly, 11 out of the 12 *japonica* breeding lines still kept the large insertion allele (Figure 5A). These results support that the temperature tolerant allele of *OsMADS51* is not associated with early flowering of temperate *japonica* varieties in the tropics and did not contribute to adaptation of temperate *japonica* rice to the tropics.

### Introgression of the Loss-of-Function Alleles of *Hd1* Might Enable Breeding of Temperate *Japonica* Varieties Adapted to the Tropical Region

Through the *japonica* rice breeding project of GUVA, the *japonica*-type breeding lines adapted to the tropics were continuously selected from the several cross combinations (Supplementary Table S2). We grew 12 breeding lines both in Korea and the Philippines. In the Philippines, all the tropical-adapted lines flowered later than common Korean *japonica* varieties during both the cropping seasons (71–81 days in WS and 74–95 days in DS) (Figures 5A,B) and these lines did not show an irregular flowering time among tillers in a single plant. Allele typing of the major heading date genes showed that all the tropical-adapted breeding lines had the functional alleles for the *OsPRR37, DTH8, Ghd7, Ehd1*, and *RFT1* genes and Type 1 promoter for *Hd3a* gene, as observed from common Korean temperate *japonica* varieties, except for *Hd1* alleles. Interestingly, all 12 tropical-adapted lines had non-functional *hd1* alleles [Type 13, Type 14, and *photoperiod sensitivity 1* (*se1*)] (Figure 5A). Furthermore, the eight Korean temperate *japonica* varieties and 12 GUVA tropical-adapted lines were compared for their average DTH. The GUVA-derived *japonica* lines exhibited quite delayed flowering (18.8 days in WS and 24.6 days in DS) compared to the Korean temperate *japonica* varieties (Figure 5B), but they flowered earlier (8.2 days) than the Korean temperate *japonica* varieties in natural LD condition in Korea. These results indicate that the functional *Hd1* alleles of temperate *japonica* varieties cause very early flowering with an irregular flowering pattern among tillers in natural SD condition. Thus, the loss-of-function alleles of *Hd1* might have been strongly selected as key genes during the breeding process and its introgression might have enabled adaptation of temperate *japonica* varieties to the tropics.

### Phylogenetic Analysis of the Rice Accessions

**FIGURE 6:**
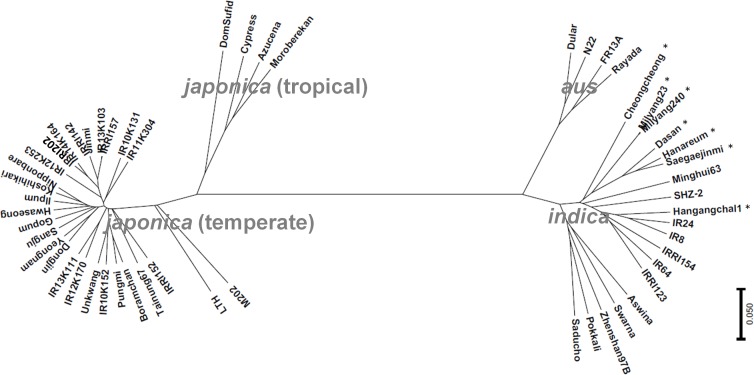
Phylogenetic analysis. All materials, except for three accessions (Kitaake, Taichung 65, and Kasalath), were analyzed by 6K SNP chip and the phylogenetic tree was constructed by using 2,918 SNP data points. The samples were grouped into *aus, indica*, tropical *japonica*, and temperate *japonica* types consistent with the previous report (McNally et al., 2009). Asterisk (^∗^) refers to Tongil-type rice cultivars.

In terms of phenotypes, all the GUVA tropical-adapted lines showed characteristics of *japonica* varieties such as short grains and sticky texture of cooked rice (data not shown). However, during the *japonica* rice breeding process, the *indica*-genome-added lines were also used as parents. To examine the genome status of the breeding lines, a phylogenetic analysis was conducted using a chromosome-wide 2,918 SNP data points. Based on the classification by McNally et al. (2009), the materials were grouped into four groups: *aus, indica*, tropical *japonica*, and temperate *japonica* types. All 12 GUVA breeding lines were clustered with the temperate *japonica* varieties including Dongjin and Nipponbare (Figure 6), indicating that they are highly close to the temperate *japonica* rice type at the genome level. This result suggested that an introgression of the non-functional alleles of *Hd1* into the temperate *japonica* background, together with a conventional plant selection breeding method (this might cause a small change in the genome of the temperate *japonica* background), enabled the breeding of *japonica* varieties for the tropics.

## Discussion

Currently, more than 40 flowering-related genes have been cloned in the rice genome and the process of gene identification and its functional studies were mostly done in controlled conditions like a growth chamber or room and/or at a paddy field in high-latitude locations (natural LD) (Lee and An, 2015a,b; Hori et al., 2016). In addition, through allele typing of major rice flowering genes from diverse collections, the major alleles/genes for the geographical adaptation/expansion, geographical favorable alleles, and diversification of alleles were studied in mostly temperate zone (Naranjo et al., 2014; Gómez-Ariza et al., 2015; Itoh et al., 2018; Ye et al., 2018) where *japonica* rice is cultivated. In terms of breeding purposes, evaluations of the genetic effects of the identified flowering genes at the natural field conditions and the further use of the proper allele types of the genes, especially those derived from the natural variations not transgenic-based identified genes, are important to optimize flowering time of local rice varieties. In this study, we examined the allele types of major seven flowering genes and DTH in three different natural environments from the 45 diverse accessions. Through this, we analyzed allele preference of each flowering gene among the rice types and the genetic effect of each gene on flowering time in the temperate and tropical locations, respectively. Furthermore, we compared DTH and allele types of flowering genes between common temperate *japonica* varieties and the 12 GUVA breeding lines and we identified the key genetic factor which contributed to adaptation of *japonica* rice to the tropics.

The *Hd1* gene was identified by fine mapping of the *Hd1* QTL derived from the Nipponbare x Kasalath cross and it was further confirmed that *Hd1* is allelic to *Se1* by the sequence analysis of the *photoperiod sensitivity 1* (*se1*) mutant having a 43-bp deletion in the first exon of *Hd1* and its background variety Ginbouzu (*Se1*) (Yano et al., 2000). The near-isogenic line (NIL) having non-functional *hd1*-Kasalath allele showed late and early flowering under controlled SD and LD conditions, respectively, compared to the background cultivar Nipponbare, indicating that *Hd1* promotes flowering under SD but inhibits it under LD (Lin et al., 2000). Takahashi et al. (2009) showed high associations between *Hd1* functionality and flowering under the controlled SD by studying the six major SD-pathway flowering genes in 64 rice germplasm accessions. In our study, a strong effect of *Hd1* on flowering promotion under the natural SD condition was clearly observed. In the 45 diverse accessions, the functional *Hd1* alleles showed quite early flowering under the natural SD condition dependent on Figure 4A, or independent of Supplementary Figure S4A, the allele types of the other flowering genes genotyped. This result means that the functional *Hd1* allele is less influenced by the other genetic factors, and is able to enforce early flowering under the SD condition.

Under an LD condition, *Hd1* represses flowering through the suppression of *Hd3a* and *RFT1* transcription. Although Hd1 is regarded as an evolutionally conserved transcriptional activator, its interaction with DTH8 directly represses *Hd3a* expression (Du et al., 2017). Alternatively, suppression of *Ehd1* by Hd1-Ghd7 complex affects transcription of florigen genes in LD (Nemoto et al., 2016). In other words, when the *Hd1* exists with the non-functional *dth8* or *ghd7, Hd1* functions as transcriptional activator for *Hd3a* or *Ehd1* respectively, resulted in flowering promotion. In consistent with above studies, the germplasm possessing the *Hd1*/*dth8* alleles (M202 and Zhenshan 97B) and the *Hd1/ghd7* alleles (Kitaake and Zhenshan 97B) showed early flowering tendency under the natural LD condition compared to the other accessions (Supplementary Figure S7). The NIL having the *hd1* allele of Kasalath and the T-DNA-inserted *hd1* mutant on the background cultivar Dongjin (*DTH8*/*Ghd7* alleles) showed significantly early flowering by 17 and 16 days, respectively, under natural LD field conditions (Lin et al., 2000; Lee et al., 2016). In our study, the single genetic effect analysis of the *Hd1* gene under the LD condition did not show a significant difference in flowering time between the *Hd1* and the *hd1* groups in both complex genotype background of flowering genes and the same genetic backgrounds (*OsPRR37*/*DTH8*/*Ghd7*/*Ehd1*/*RFT1*/*Hd3a*) (Figure 4A). However, in the closer genetic backgrounds between Korean temperate *japonica* varieties and the 12 GUVA lines, the *hd1* plants (GUVA lines) significantly flowered early (∼8.2 days) under the natural LD condition (Figure 5B). These results suggest that the function of *Hd1* under LD is influenced by other genetic factors in addition to known *DTH8* and *Ghd7*. In conclusion, the functionality of *Hd1* might be one of the major domestication traits in the tropical area whereas *Hd1* with some other genetic factors might be co-selected in the temperate zone during domestication or breeding process.

All 12 GUVA tropical-adapted *japonica* breeding lines possessed the *hd1* alleles without changes of functionality for the other flowering genes compared to Korean common *japonica* varieties. This data clearly support that *Hd1* causes too early flowering of temperate *japonica* varieties in the tropics and the introgression of *hd1* contributed for breeding of temperate *japonica* rice to the tropics. Six out of the 12 GUVA breeding lines had the *se1* allele (Figure 5A). The Korean *japonica* cultivar Jinmi harboring the *se1* allele was used as the immediate parent of the IRRI 142 and IR14K164 lines (Supplementary Table S2). The intermediate breeding lines having the Jinmi genome segments were also used for development of 10 GUVA breeding lines, resulted in a high-frequency introgression of the *se1* allele in the GUVA breeding lines. However, other non-functional *hd1* alleles, Type 13 and Type 14, were also selected during conventional breeding process although Jinmi or Jinmi-derived lines were highly used. This result supports that any types of *hd1* alleles will be effective for the breeding of temperate *japonica* rice varieties for the tropics.

Testing and adaptation of temperate *japonica* varieties in low latitudes are less studied. A successful adaptation of a *japonica* variety in Taiwan was achieved through the breeding of Taichung 65 and since then, this variety was highly utilized for the development of other Taiwanese *japonica* varieties (Hsing, 2014). A previous phylogenetic analysis constructed by using 119 simple sequence repeat markers showed that most Taiwan *japonica* varieties including Taichung 65 and Tainung 67, which were also used in this study, were clustered into Japanese temperate *japonica* varieties (Lin et al., 2012). Consistently, the genome-wide 2,918 SNP markers analysis in this study indicated that the genome status of Tainung 67 is highly close to those of Japanese and Korean temperate *japonica* varieties (Figure 6), confirming that the varieties having temperate *japonica* genomes have successfully adapted to tropical and subtropical regions. A genetic analysis by Doi et al. (2004) revealed that the non-functional allele of *Ehd1* (also called *Early flowering 1* (*Ef1*)) of Taichung 65 in combination with the *hd1* delayed flowering in an SD condition. Similarly, the *ehd1* (*ef1*) allele markedly delayed flowering when it coexisted with the *hd1* allele in a controlled SD condition (Nishida et al., 2002). The *ehd1* allele was frequently found in Taiwanese *japonica* rice varieties including Tainung 67 but not in other *japonica* and *indica* varieties (Saito et al., 2009; Takahashi et al., 2009; Wei et al., 2016), suggesting that the *ehd1* allele caused long basic vegetative growth periods in low-latitude regions. Similar to the previous reports, only two Taiwanese varieties (Taichung 65 and Tainung 67) possessed the *ehd1* allele in our diverse panel. Thus, we compared the flowering time among three temperate *japonica* types (Korean, GUVA-developed, and Taiwanese). Taiwanese cultivars showed slightly delayed flowering compared to the GUVA lines in the Philippines. However, the GUVA lines (*hd1*/*Ehd1*) showed quite delayed flowering compared to the Korean *japonica* cultivars (*Hd1*/*Ehd1*) (Figure 5B), suggesting that the functional *Ehd1* of the GUVA lines did not cause extremely early flowering in the natural SD condition. As another supporting examples in our study, the varieties having the *hd1*/*Ehd1* alleles like the GUVA lines showed late flowering at the IRRI field (Figure 3). To conclude, the photoperiod insensitivity of Taichung 65 might have been achieved firstly by the introgression of the *hd1* allele and then the introduction of the *ehd1* allele for further delaying of flowering. The *ehd1* allele needs to be tested in the GUVA line background for further yield enhancement through extending duration of vegetative growth.

In contrast to the adaptation of temperate *japonica* varieties to the tropics, the *indica* germplasm was well-utilized to breed high-yielding rice varieties (called as Tongil type rice) under the temperate climate in Korea. Since the 1970s, many Tongil-type varieties were used as rice varieties or breeding materials in several Asian countries (Kim et al., 2012; Dingkuhn et al., 2015) because of their wide geographical adaptability. In this study, most of the tropical-origin varieties showed normal flowering (89–116 days) in Suwon, Korea (Figure 3), suggesting that the flowering time of the *indica* germplasm may not be a major constraint for breeding of *indica* type varieites for the temperate zones. The genome status of all seven Tongil-type varieties was highly close to the *indica* genome (Figure 6) although a *japonica* variety was used as one of the parents. Interestingly, all seven Tongil varieties and all four IRRI *indica* varieties (IR8, IR24, IRRI 123, and IRRI 154) had the non-functional *Hd1*-Type 7 allele (Figure 3). This result suggests that the *Hd1*-Type 7 of Tongil varieties might have originated from IR8 or IR24 (called the Green Revolution rice varieties) because these two IRRI varieties were frequently used as donors for the breeding of Tongil-type varieties (Kim et al., 2012). This non-functional *hd1* allele might also have contributed to a wide geographical adaptation of the Tongil varieties to tropical and temperate regions.

DTH8 is known as a flowering repressor under LD conditions and its function is unclear under SD conditions (Wei et al., 2010; Yan et al., 2011; Du et al., 2017). The functionality of *DTH8* did not significantly influence flowering time in the natural LD condition in the single gene effect analysis (Supplementary Figure S4C), and when the *DTH8* and the *dth8* alleles were compared in the same genotype background (*hd1/OsPRR37/Ehd1*/*RFT1*/*Ghd7*) (Figure 4B). In contrast, in the natural SD condition, the plants with the *dth8* allele showed delayed flowering (2–11.5 days) compared to the ones with *DTH8* in *hd1* backgrounds (Figure 4B), suggesting that *DTH8* may promote flowering in the absence of *Hd1* in the SD condition. Consistent with our results, the genetic effect of *DTH8* might be influenced by other genetic factors including *Hd1* (Lin et al., 2003; Yan et al., 2011).

*OsPRR37* and *Ghd7* are flowering repressors in LD conditions (Hori et al., 2016; Nemoto et al., 2016). The non-functional alleles for these genes were rare alleles found only in five and two accessions, respectively, in the diversity panel (Figure 2). These non-functional alleles also co-existed with the non-functional alleles of another flowering genes (Figure 3). However, the single genetic factor analysis showed that both non-functional alleles of *OsPRR37* and *Ghd7* were involved in the early flowering tendency in all three conditions (Supplementary Figure S4), especially in the natural LD condition. In the case of *OsPRR37*, the varieties having the non-functional allele types (Kitaake, Zhenshan 97B, Dular, and Saducho), except Kasalath, flowered early in the natural LD condition (Supplementary Figure S7). Kitaake and Zhenshan 97B having the *ghd7* and *osprr37* exhibited very early flowering in the natural LD condition (Supplementary Figure S7). Consistently, the *japonica* varieties having non-functional alleles of both genes flowered extremely early under a natural LD condition because of the additive genetic effect of the genes (Kim et al., 2013) and these allele types might have contributed to adaptation of rice to the northernmost regions of rice cultivation (∼53° N) (Koo et al., 2013). In the single gene effect analysis using the T-DNA-inserted mutants in the Dongjin background, the *ghd7* and *osprr37* mutants flowered earlier by 34 and 7 days, respectively, than Dongjin in a natural LD field condition (Koo et al., 2013; Lee et al., 2016), suggesting that the *Ghd7*-*Ehd1*-*Hd3a*/*RFT1* pathway is a major flowering pathway in natural LD conditions.

In the regulatory region of *Hd3a*, a large mobile DNA sequence (∼4.9 kb) was inserted at the -292 bp position from the translation start codon (ATG) in *Hd3a*-NT2 allele (Supplementary Figures S1, S2). Probably, the large insertion did not cause the non-expressed *Hd3a* allele because another florigen gene *RFT1* is already non-functional in Zhenshan 97B. However, the influences of the large insertion to *Hd3a* transcription need to be tested in the future. Recent studies of the regulatory elements for *Hd3a* expression characterized several *cis*-elements at different positions on *Hd3a* promoter. Pasriga et al. (2018) identified the regulatory region for vascular tissue-specific expression of *Hd3a* (-397 to -197 region from ATG) using *GUS* reporter system and Du et al. (2017) identified two *cis*-elements of *Hd3a* (-1,475 to -1,274 and -526 to -313 regions from ATG) for Hd1 binding using yeast one-hybrid assay. Another report by Goretti et al. (2017) identified *CO Response Element 2* (*CORE2*) (-176 to -168 from ATG) using electrophoretic mobility shift assay. This new *Hd3a*-NT2 allele will be useful material to study a regulatory element of *Hd3a* promoter.

Under the natural LD condition in Suwon, three *indica* varieties (Swarna, Aswina, and Pokkali) and two *aus* varieties (Rayada and FR13A) did not flower before frost winter (> 180 days) (Figure 3). No flowering was observed in a natural LD condition when rice plants had the *ehd1*/*rft1* (Zhao et al., 2015) or the *Hd1*/*rft1* alleles combination (Ogiso-Tanaka et al., 2013). Two accessions (Pokkali and Aswina) possessing *Hd1*/*rft1* are expected for no flowering based on the above research but the others had *Hd1/RFT1* (FR13A and Rayada) or *hd1/rft1* (Swarna) alleles (Figure 3). In the cases of FR13A and Rayada, Hd1-mediated flowering suppression (Hd1-DTH8 complex and Hd1-Ghd7 complex via *Ehd1* suppression) will be functional, suggesting that transcriptional defect of *RFT1* or unknown strong flowering repressor are involved in no flowering in LD. In contrast, Swarna had both non-functional *hd1/rft1* alleles, assuming presence of unknown strong repressor of *Hd3a*. Interestingly, all these five no flowering accessions showed the latest flowering even in the SD conditions, especially in WS (Figure 3). These five accessions and Dom Sufid had unique Type 4 or Type 6 promoters of *Hd3a*. The relationship between *Hd3a* types and late flowering under SD needs to be studied in the future.

All the 45 tester accessions and 12 GUVA *japonica* breeding lines showed early flowering in the natural SD conditions (Figures 3, 5A). This result supports the previous finding that a conventional SD treatment like placing pots containing rice plants into a dark room may be effective to promote flowering and, in turn, to shorten the duration of a breeding cycle. In the natural SD conditions at IRRI, most of the accessions flowered earlier in WS than in DS (Supplementary Figure S8). Around 30–40 days from seeding (basic vegetative growth phase), the day-length is slightly decreasing at IRRI during WS whereas increasing during DS, although the actual day-length on the seeding date is longer in WS than in DS (Figure 1F). This observation suggests that the rice plants are continuously monitoring a minor change of day-length (becoming shorter) not the absolute day-length (h) although most of days during both cropping seasons are close to the SD condition. Similarly, in the natural LD condition in Suwon, rice plants might perceive shorter day-lengths in early July (the absolute day-length is the longest), and trigger the flowering pathway genes. In contrast, the few accessions having the *Hd3a*-Type 6 allele flowered slightly late in DS than in WS (Figure 3). However, the influences of some other environmental factors such as daily radiation and temperature as well as those of other genetic factors such as the *Hd3a*-Type 6 allele need to be examined to elucidate the biological/genetic mechanisms causing a minor flowering difference between the two cropping seasons at the same location.

Many genetic factors regulating flowering have been identified at the gene level. Through marker-assisted selection, the putative favorable alleles of the identified genes can be tested and utilized to adjust flowering in elite rice varieties, which can improve yields, cropping systems, and farmer’s income. Here, the association of *Hd1* with the adaptation of *japonica* varieties in the tropical regions was examined. For further improvement of the GUVA lines by extension of vegetative growth phase, non-functional alleles for *Ehd1* and *DTH8* loci are needed to be tested in the future.

## Author Contributions

S-RK, U-SY, O-YJ, and J-SL conceived the research. GT and M-RY, JK, and C-KL designed and conducted field experiments in the Philippines and Korea, respectively. S-RK, I-RC, and J-SL conducted allele typing of rice flowering genes. GP performed phylogenetic analysis. S-RK, I-RC, KJ, and J-SL analyzed the data and wrote the manuscript. All authors read and approved the manuscript.

## Conflict of Interest Statement

The authors declare that the research was conducted in the absence of any commercial or financial relationships that could be construed as a potential conflict of interest.

## Footnotes

1 http://www.knowledgebank.irri.org

2 http://www.appliedbiosystems.com/

3 http://dna.macrogen.com/

4 https://blast.ncbi.nlm.nih.gov/

5 http://www.mbio.ncsu.edu/BioEdit/

6 http://gsl.irri.org/
