# Supplementary material for: Loss-of-Function Alleles of Heading date 1 (Hd1) Are Associated With Adaptation of Temperate Japonica Rice Plants to the Tropical Region
Source: Front Plant Sci. 2018 Dec 10;9:1827. doi: 10.3389/fpls.2018.01827 (PMC6295564; doi:10.3389/fpls.2018.01827)
Supplement: Supplementary file 1 [file Presentation_1.pdf]

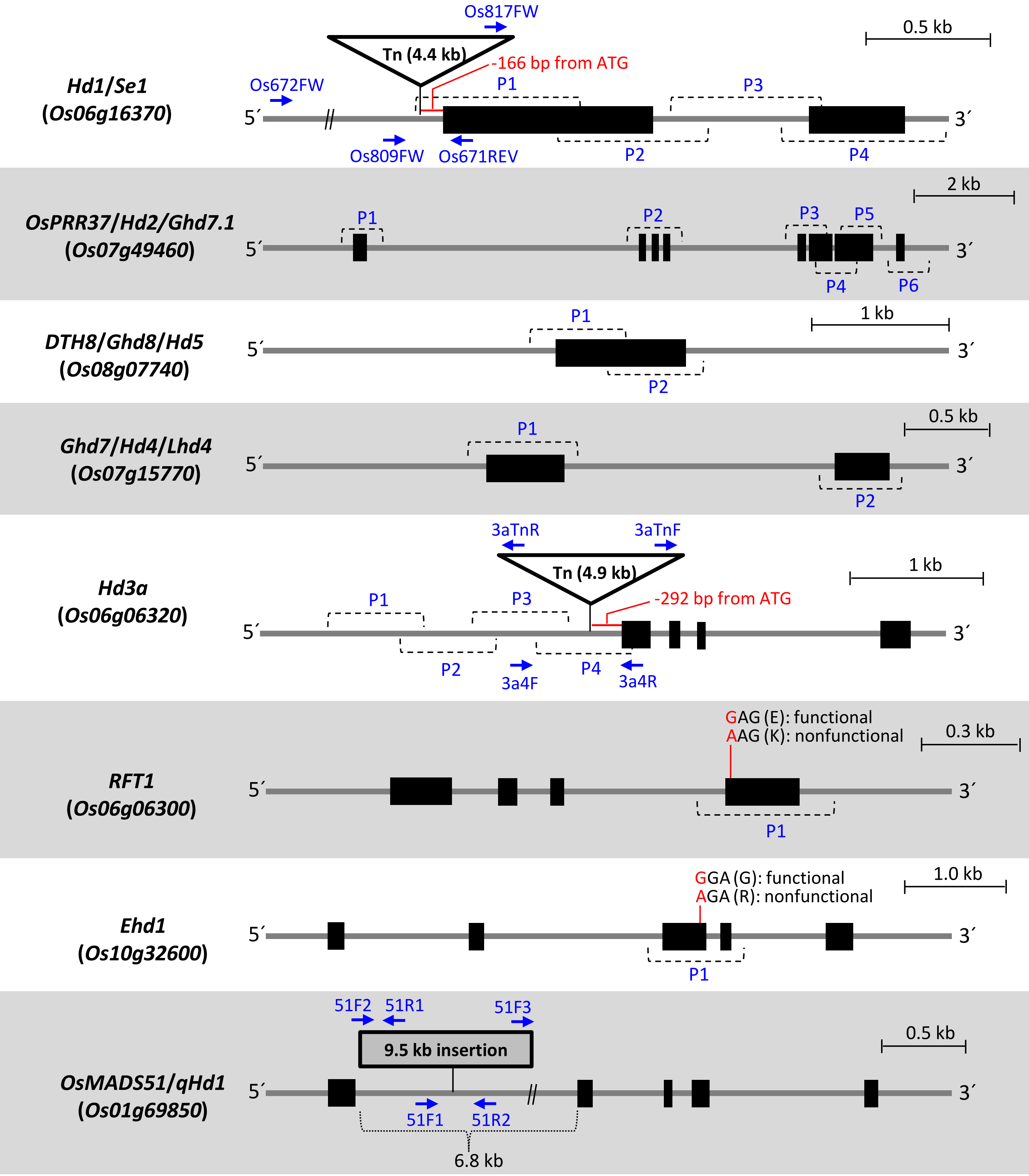

Supplementary Figure S1. Genomic structures of the major rice flowering genes tested in this study. To each gene, another gene/QTL names with MSU gene ID are co-presented. Filled box means protein coding sequences (CDS). PCR-sequencing regions in each gene are depicted with the primer (p) sets and the primer information are shown in the Supplementary Table S3. Primers for detection of a large insertion are presented by blue arrows.

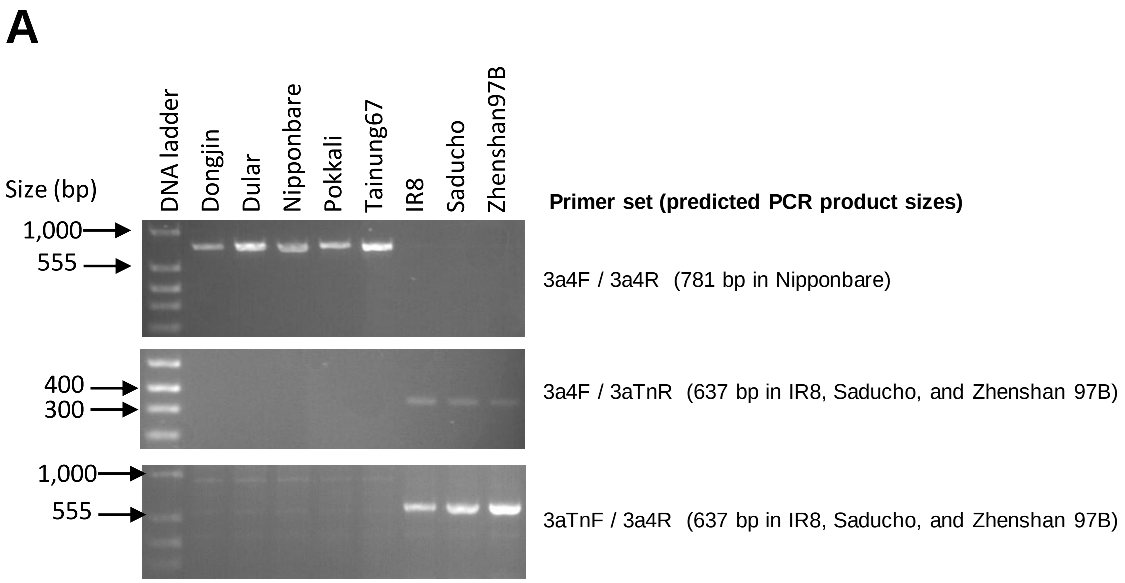

B

AGCGAGGGTGAATGTGTGATTTGATGGGTTTTTCCACGTTAAATTCGTGTGTCTCCGCT
GTGATTGTTTTTCGTTTACTTCATATTTCTATACGTCGTTCATAACAAGATGATATTATTC
TTCTGCAGCTAAATTAAAGTGAAAGTTGGACATGGACATGGACATAGTAATTTTGCATG
GCCATCATCTTGCCCTCCTATATAAAGCGGCCATCTCACTCTCAACCACAGCTCGATCCAT
CAGCCCTGCACCACACACAGTTCAGCTAGCAGATCACCTAGCTAGATAGCTGCCTCTATC
ACAGTATATTTGCTCCCTGAAACTTGCTGCTGCTGCAATAGCTAGCAGCTGCAGCTAGTA
AGCAAAACTATATACCTTCAGGGTTTTTTGCAAGATCG**ATG (*Hd3a* Start codon)**

Supplementary Figure S2. A new allele of *Hd3a* promoter, *Hd3a*-NT2 allele. The *Hd3a*-NT2 allele was identified by sequence analysis of the chromosome 6 sequence of IR8 (GenBank accession no. CM007601.1). Further, the structure of *Hd3a*-NT2 promoter was confirmed by PCR amplifications surrounding the transposable element (~4.9 kb) insertion site of the *Hd3a* promoter region (A) and by Sanger sequencing of PCR products amplified by 3aTnF/3a4R primer set from Zhenshan 97B (B). The underlined sequence (292 bp) can be aligned with the common *Hd3a* promoter sequence and the bold sequence is a part of the mobile DNA element. The gene structure of *Hd3a* with primer locations are depicted in the Supplementary Figure S1.

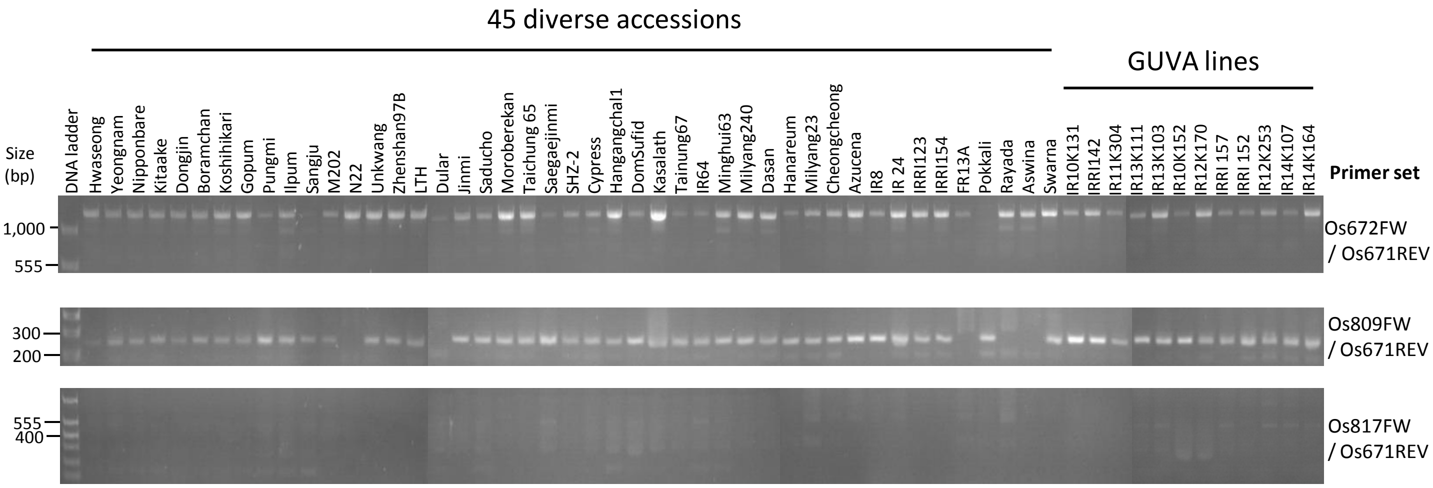

Supplementary Figure S3. PCR amplification of *Hd1* promoter region to identify presence/absence of the mobile DNA element (~4.4 kb) which caused the non-expressed *hd1* allele using the diagnostic primer sets (Goretti et al., 2017). The order of samples is consistent with that of FIGURE 3 and FIGURE 5. The gene structure of *Hd1* with the primer locations are depicted in the Supplementary Figure S1.

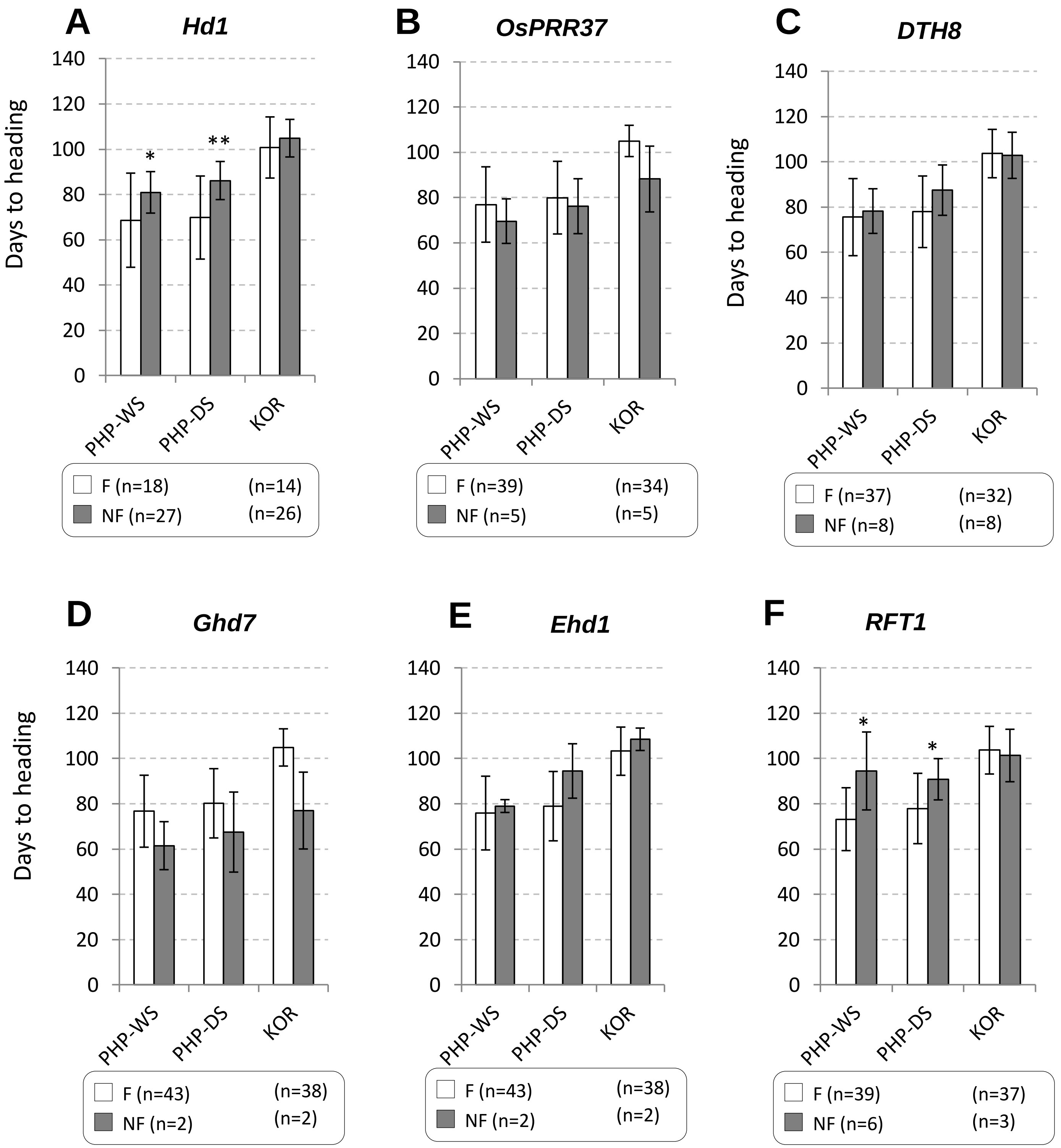

Supplementary Figure S4. Genetic effects of the major heading date genes on flowering time. Forty-five accessions were divided into two groups based on the gene functionality (F, functional alleles and NF, non-functional alleles) and the mean values of DTH was calculated between two groups in three different environments. Number of samples is presented in the parentheses. DTH data obtained in Korea excluded the non-flowering five accessions (FR13A, Pokkali, Rayada, Aswina, and Swarna). PHP, Philippines; KOR, Korea. DS, dry season; WS, wet season. Asterisks represent a significant difference between two groups based on Student's *t*-test (* $\alpha = 0.05$ and ** $\alpha = 0.01$).

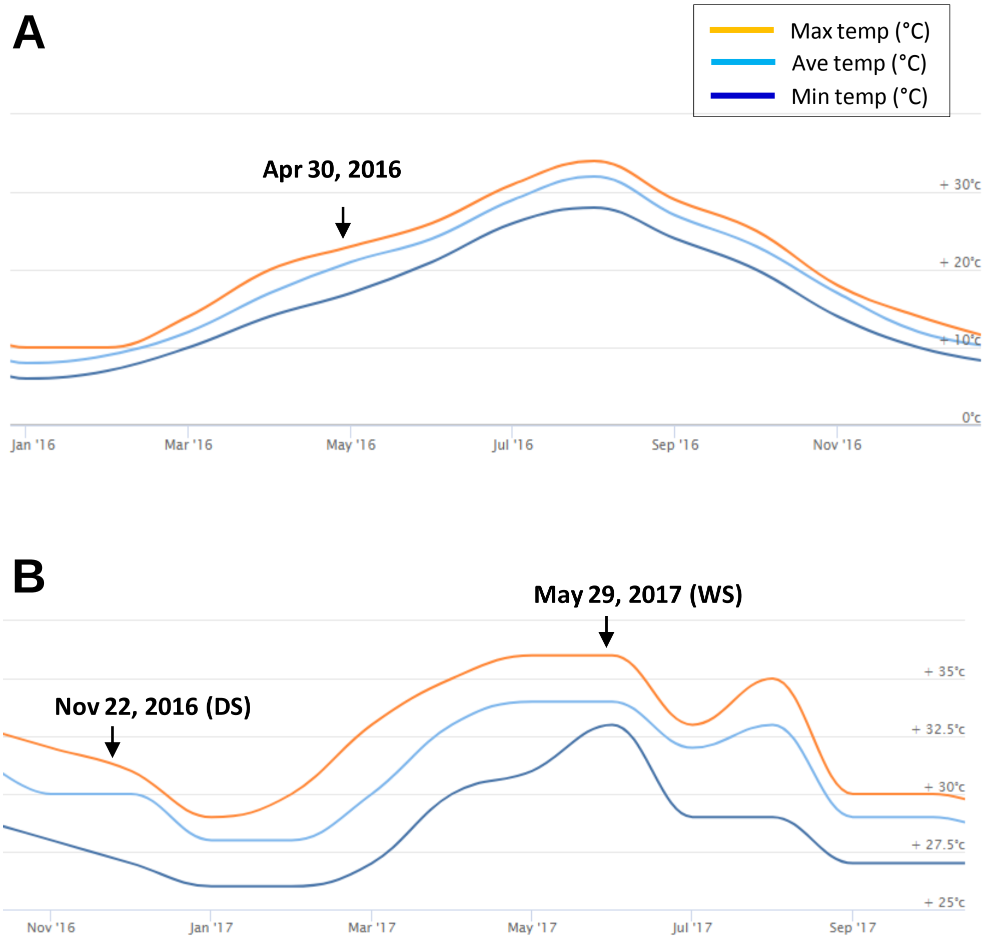

Supplementary Figure S5. Temperature data during experiments in Suwon, Korea (A) and Los Baños, the Philippines (B). Seeding date of each cropping season is inserted in the figure. The temperature data was obtained from the World Weather Online (https://www.worldweatheronline.com/).

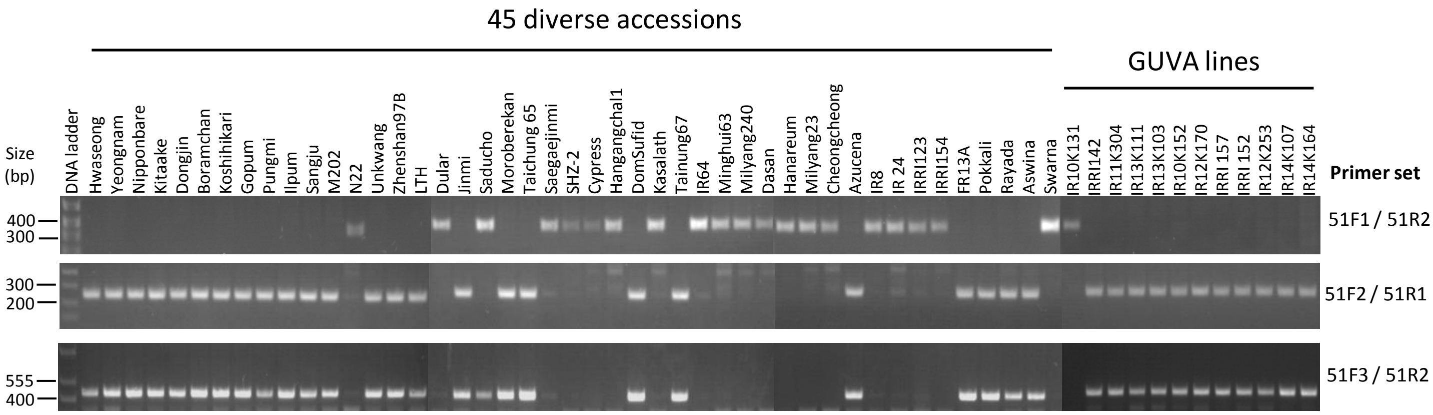

Supplementary Figure S6. PCR amplification of a large sequence insertion (~9.5 kb) at the 1st intron of *OsMADS51* gene. The order of samples is consistent with that of FIGURE 3 and FIGURE 5. The gene structure of *OsMADS51* with primer locations are depicted in the Supplementary Figure S1.

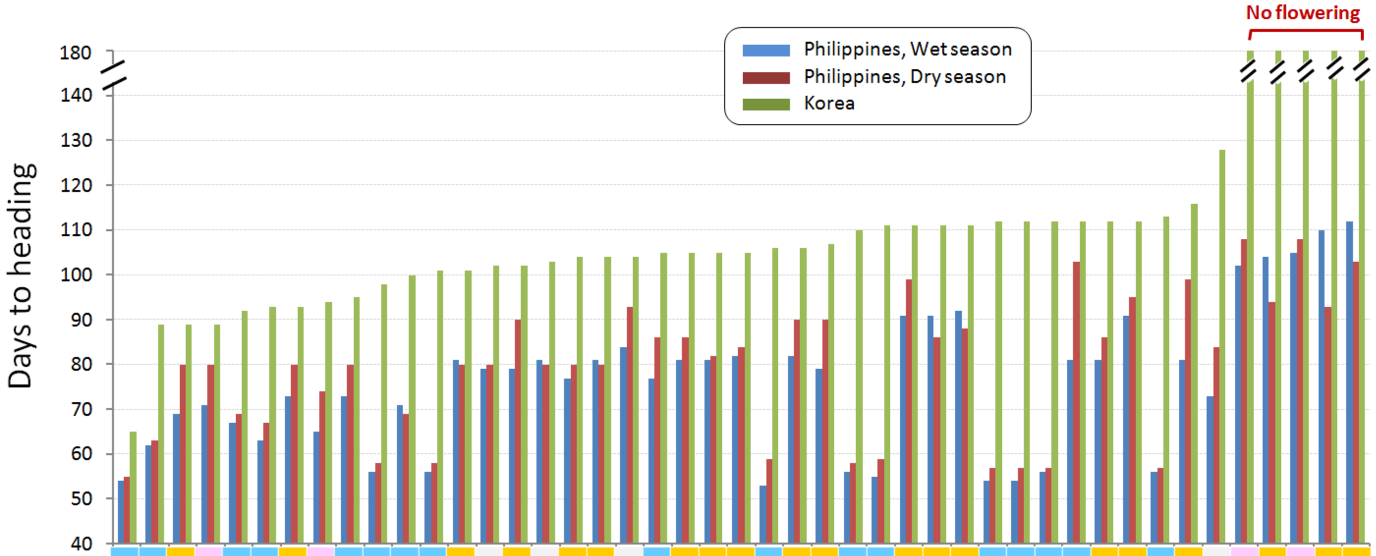

Variety	Rice type	Hd1	OsPRR37	DTH8	Ghd7	Ehd1	RFT1	Hd3a
Kitaake	j-te	GBZ	H02	T1	G-0a	G	E	T1
Sangju	j-te	GBZ	H13	T1	G-2	G	E	T1
Zhenshan97B	ind	T01	H06	ZS	G-0	G	K	NT2
Dular	aus	T13	H24	T3	G-2	G	E	T3
Unkwang	j-te	T14	H13	T1	G-2	G	E	T1
M202	j-te	GBZ	H13	T8	G-2	G	E	T1
Saducho	ind	T07	H06	T8	G-1	G	E	NT2
N22	aus	T13	H07	T3	G-2	G	E	T3
Jinmi	j-te	se1	H11	T1	G-2	G	E	T1
Pungmi	j-te	T06	H05	T1	G-2	G	E	T1
LTH	j-te	T09	H05	T1	G-2	G	E	NT1
Koshihikari	j-te	T06	H13	T1	G-2	G	E	T1
Dasan	ind	T07	H07	T1	G-1	G	E	T1
Cypress	j-tr	T13	H07	T1	G-2	G	E	T1
Hangangchal1	ind	T07	H07	T8	G-1	G	E	T1
DomSufid	j-tr	NT2	H18	T4	G-2	G	K	T6
Saegaejinmi	ind	T07	H07	T1	G-1	G	E	T1
Hanareum	ind	T07	H07	T1	G-1	G	E	T1
Azucena	j-tr	T13	H18	T2	G-2	G	E	T1
Taichung65	j-te	T65	H05	T1	G-2	R	E	T1
Kasalath	ind	T13	H24	T4	G-7	G	E	T3
Milyang240	ind	T07	H07	T1	G-1	G	E	T1
Milyang23	ind	T07	H07	T1	G-1	G	E	T1
Hwaseong	j-te	GBZ	H05	T1	G-2	G	E	T1
Cheongcheong	ind	T07	H07	T6	G-1	G	E	T1
SHZ-2	ind	T07	H07	T8	G-4	G	E	T1
Gopum	j-te	T06	H13	T1	G-2	G	E	T1
Dongjin	j-te	GBZ	H13	T1	G-2	G	E	T1
IR24	ind	T07	H07	T8	G-1	G	E	T1
IRRI123	ind	T07	H09	T3	G-1	G	E	T1
IRRI154	ind	T07	H09	T3	G-1	G	E	T1
Yeongnam	j-te	GBZ	H13	T1	G-2	G	E	T1
Nipponbare	j-te	T06	H13	T1	G-2	G	E	T1
Boramchan	j-te	GBZ	H11	T1	G-2	G	E	T1
Tainung67	j-te	T65	H05	T1	G-2	R	E	T1
IR64	ind	T13	H07	T6	G-1	G	E	T1
IR8	ind	T07	H07	T8	G-1	G	K	NT2
Ilpum	j-te	GBZ	H05	T1	G-2	G	E	T1
Minghui63	ind	T07	H07	T8	G-1	G	E	T1
Moroberekan	j-tr	T13	NT1	T2	G-2	G	E	T1
FR13A	aus	T08	H18	T4	G-7	G	E	T4
Pokkali	ind	T01	H07	T4	G-6	G	K	T6
Rayada	aus	T08	H18	T4	G-7	G	E	T4
Aswina	ind	T08	H07	T3	G-1	G	K	T6
Swarna	ind	T07	H07	T5	G-1	G	K	T6

Supplementary Figure S7. DTH and the genotypes of the major seven flowering genes from 45 diverse accessions. Samples were sorted by DTH of the natural LD condition in Suwon, Korea.

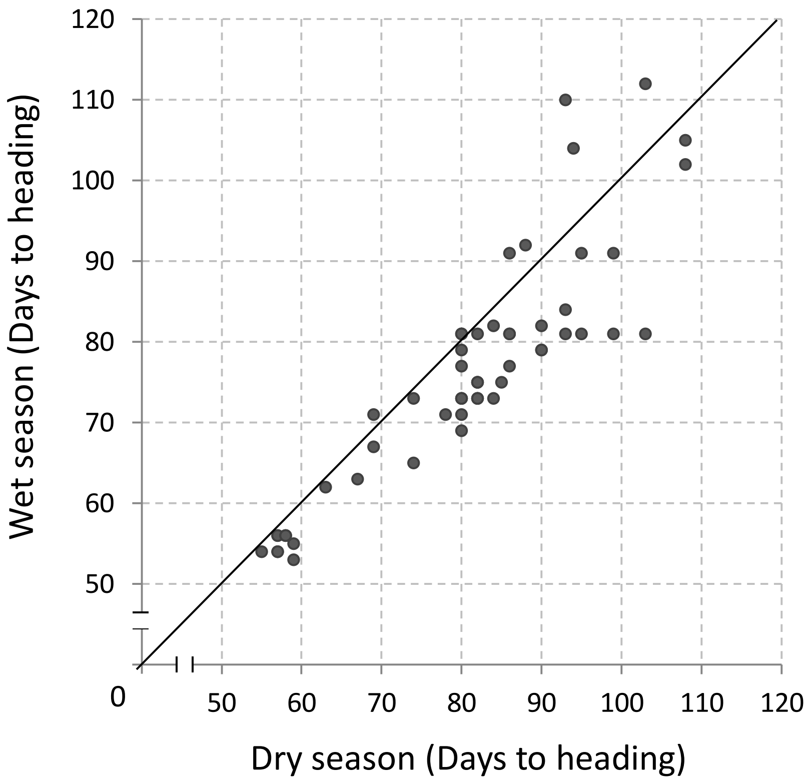

Supplementary Figure S8. Comparison of DTH between DS and WS. DTH data collected at IRRI from the 57 accessions (45 tester accessions and 12 GUVA breeding lines) in the both cropping seasons (DS and WS) were plotted.

Supplementary Table S1. Rice accessions and flowering time in the three different conditions

Variety	Origin	Rice type	Days to heading (DTH)		
			Los Baños (DS)	Los Baños (WS)	Suwon
FR13A	India	aus	108	102	No flowering
Rayada	Bangladesh	aus	108	105	No flowering
N22	India	aus	74	65	94
Dular	India	aus	80	71	89
Saducho	S. Korea	ind	80	73	93
Zhenshan97B	China	ind	80	69	89
SHZ-2	China	ind	90	79	107
Minghui63	Taiwan	ind	99	81	116
IR8	Philippines	ind	95	91	112
IR24	Philippines	ind	99	91	111
IRRI123	Philippines	ind	86	91	111
IRRI154	Philippines	ind	88	92	111
IR64	Philippines	ind	86	81	112
Kasalath	Bangladesh	ind	86	81	105
Aswina	Bangladesh	ind	93	110	No flowering
Pokkali	India	ind	94	104	No flowering
Swarna	India	ind	103	112	No flowering
Saegaejinmi	S. Korea	ind*	80	77	104
Hangangchal1	S. Korea	ind*	90	79	102
Milyang240	S. Korea	ind*	82	81	105
Dasan	S. Korea	ind*	80	81	101
Hanareum	S. Korea	ind*	80	81	104
Milyang23	S. Korea	ind*	84	82	105
Cheongcheong	S. Korea	ind*	90	82	106
Moroberekan	Guinia	j-tr	84	73	128
Cypress	United States	j-tr	80	79	102
Azucena	Philippines	j-tr	93	84	104
DomSufid	Iran	j-tr	80	81	103
Hwaseong	S. Korea	j-te	59	53	106
Yeongnam	S. Korea	j-te	57	54	112
Dongjin	S. Korea	j-te	59	55	111
Boramchan	S. Korea	j-te	57	56	112
Ilpum	S. Korea	j-te	57	56	113
Sangju	S. Korea	j-te	63	62	89
Gopum	S. Korea	j-te	58	56	110
Pungmi	S. Korea	j-te	58	56	98
Unkwang	S. Korea	j-te	69	67	92
Jinmi	S. Korea	j-te	80	73	95
Nipponbare	Japan	j-te	57	54	112
Koshihikari	Japan	j-te	58	56	101
Kitaake	Japan	j-te	55	54	65
LTH	China	j-te	69	71	100
M202	United States	j-te	67	63	93
Taichung 65	Taiwan	j-te	86	77	105
Tainung67	Taiwan	j-te	103	81	112

ind: *indica*, j-tr: tropical *japonica*, j-te: temperate *japonica*; *: Tongil type *indica*

Supplementary Table S2. The japonica breeding lines/varieties derived from the GUVA project for the tropics

IRRI designation	Variety name (NSIC number)[a]	Cross (female/male)	Year of cross	Year of development[b]
IRRI 142	MS 11 (NSIC Rc 170)	Jinmi/Cheolweon 46	1993	2008
IRRI 152	Japonica 1 (NSIC Rc 220)	IR77863-95-2-3/IR71667-19-4-2-4	2003	2009
IRRI 157	Japonica 2 (NSIC Rc 242)	IR80091-46-2-1/IR71663-14-2-3-5	2004	2011
IR10K131		IR 71667-19-4-2-4/IR 79037-7-2-2	2005	2010
IR10K152		HR24580-15-1/IR03K105	2007	2010
IR11K304		IR07K125//IR 83265-1-1-13-26-3-1/IR05K109	2007	2011
IR12K170		IR10K128/IR81219-13-3-1-3	2008	2012
IR12K253		IR07K150/IR 84399-58-3-1	2009	2012
IR13K111		IR07K142/IR84233-11-3-3//IR07K142	2009	2013
IR13K103		IR07K142/IR84233-11-3-2//IR07K142	2009	2013
IRRI 202	Japonica 6 (NSIC Rc 484)	IR68333-R-R-B-22/IR86743-28-1-4	2010	2017
IR14K164		Jinmi/IR86088-52-1-2	2010	2014

[a]Variety name was given by the National Seed Industry Council (NSIC) of Philippines.
[b]In case of the lines became the varieties in the Philippines, the year of variety registration was presented.

Supplementary Table S3. Primers list used in this study

Gene	Primer set	Forward primer (5 -> 3)	Reverse primer (5 -> 3)	PCR product (bp) in IRGSP1.0
Hd1	P1	TTCCCCTCCCTAGCTCCTTCCAA	CGGTTGTCGTAGTACGAATTGTAC	785
	P2	ACGAGGAGGTGGACTCTTG	ATCGGTTCCATTTAATCAGCCT	678
	P3	CAGAGAATGAACATCTATTACTG	CAGGATTCTGGAATTTGGCAT	581
	P4	GAAAGACCTCATGAAAAGTAGG	GCTATCCGGAAATTACAAAGCA	768
OsPRR37	P1	GAGATTGATTTGCACAACTGC	TGCTTGCTTATCGTCCTTGT	746
	P2	CGGCTTCTTTGTTAAGGACA	TGCCTTCAGAAATCATTGTT	1,062
	P3	TGTGTTGCTGTCAAGTTCTCTT	GCACTTTGGAGGAGCAATTA	784
	P4	CTCCTAGAAATTTAAACACAGCT	CTGCATTGTTAGCCACTTCA	810
	P5	GTCAAACTCAGATGCTGCAC	CCCGATCCCATTTAGCTAGTC	820
	P6	CTCTTGAAACACTGTCCTTTAC	GCTGCCAGACATGGACGCAAATCT	777
DTH8	P1	GCTTTGTGTCCGCATCGATACCGTCT	AGCGGGTAATGCCCGTCGATGAC	752
	P2	CGACAAGTGCCAGCGCGAGAAGC	GCCATGGGCCAAACTACACATATC	782
Ghd7	P1	AGCTGATCGAGCTCAAGTGAC	GGCAGCAGAAATGAAGAGTTG	732
	P2	TTTGCTTATGCGTACATCTGG	ATGCATGATGATCAGTCATATATAGTC	489
Hd3a	P1	CGCCGACATAGAAAGGAAAG	AACCGGTCAACTAACGGAAA	789
	P2	TGATCAAGCATATATTCAAAGTCAA	TCATATTTGTTGCTAATTTGTTGG	779
	P3	AACTAACGGTACGGAAATGGT	TTCCTGTACGTGTGGACGAG	770
	P4	AACTACGACGTCGACTGCTG	GCACCAACTACGACACATGG	748
	3a4F / 3a4R	ACTGTACTGTAGCTAGATTACGCT	CACATACAAGGGTGTAGAATGTCC	781
	3a4F / 3aTnR	ACTGTACTGTAGCTAGATTACGCT	TCCGCTCCGCTTCGTCAG	-
	3aTnF / 3a4R	AGTGAGATTGTTGCTGGCTG	CACATACAAGGGTGTAGAATGTCC	-
RFT1	P1	CAGATTTGAAGGATAGGGCT	CACACTTAAGAGCCTGCATG	451
Ehd1	P1	GCTCTAGTGAAGTGTTCGAG	TCTGGAGGGAATTTGCCCTT	939
OsMADS51	51F1 / 51R2	CGACATTAACAATGTGAAGTGC	CTCCATAAAACACCGGTCATG	11,094
	51F2 / 51R1	GTCAAACATGCAAGCAAGGATG	GTGAACTACAGGTACGCTATG	257
	51F3 / 51R2	AGCAACTCCTACATAGCCTCA	CTCCATAAAACACCGGTCATG	465

Note: In some accessions, PCR amplifications with above primer sets were not good. So, we designed additional primers for clear amplification but those primers were not presented in this Table.
